# Global profiling of prolactin-modulated transcripts in breast cancer *in vivo*

**DOI:** 10.1186/1476-4598-12-59

**Published:** 2013-06-12

**Authors:** Takahiro Sato, Thai H Tran, Amy R Peck, Chengbao Liu, Adam Ertel, Justin Lin, Lynn M Neilson, Hallgeir Rui

**Affiliations:** 1 Department of Cancer Biology, Kimmel Cancer Center, Thomas Jefferson University, Philadelphia, PA, USA

**Keywords:** Breast cancer, Prolactin, Stat5

## Abstract

**Background:**

Prolactin (PRL) is essential for normal mammary gland development. PRL promotes mammary tumor formation in rodents and elevated serum prolactin is associated with increased risk of estrogen-receptor positive breast cancer in women. On the other hand, PRL may also exert pro-differentiation effects and act to suppress invasive features of established breast cancer. Previously published limited global transcript profiling analyses of prolactin-regulated gene expression in human breast cancer cells have exclusively been performed *in vitro*. The present study aimed to shed new light on how PRL modulates estrogen receptor (ER)-positive breast cancer through global transcript profiling of a human breast cancer xenograft model *in vivo*.

**Methods:**

The prolactin-responsive human T47D breast cancer cell line was xenotransplanted into nude mice and global transcript profiling was carried out following treatment with or without human PRL for 48 h. A subset of PRL-modulated transcripts was further validated using qRT-PCR and immunohistochemistry.

**Results:**

The *in vivo* analyses identified 130 PRL-modulated transcripts, 75 upregulated and 55 downregulated, based on fold change >1.6 and P-value <0.05. From this initial panel of transcripts, a subset of 18 transcripts with established breast cancer-relevance were selected and validated by qRT-PCR. Some but not all of the transcripts were also PRL-modulated *in vitro*. The selected PRL-modulated transcripts were tested for dependence on Stat5, Jak1 or Jak2 activation, and for co-regulation by 17β-estradiol (E2). The protein encoded by one of the PRL-regulated transcripts, PTHrP, was examined in a panel of 92 human breast cancers and found by *in situ* quantitative immunofluorescence analysis to be highly positively correlated with nuclear localized and tyrosine phosphorylated Stat5. Gene Ontology analysis revealed that PRL-upregulated genes were enriched in pathways involved in differentiation. Finally, a gene signature based on PRL-upregulated genes was associated with prolonged relapse-free and metastasis-free survival in breast cancer patients.

**Conclusions:**

This global analysis identified and validated a panel of PRL-modulated transcripts in an ER-positive human breast cancer xenotransplant model, which may have value as markers of relapse-free and metastasis-free survival. Gene products identified in the present study may facilitate ongoing deciphering of the pleiotropic effects of PRL on human breast cancer.

## Background

Prolactin (PRL) is a pituitary hormone that is critical for normal mammary gland development by promoting proliferative expansion of the secretory alveolar cell compartment during pregnancy and for terminal differentiation of these milk-producing cells during lactation. Prolactin is also strongly implicated in breast cancer. On one hand, accumulating evidence suggests that PRL promotes breast cancer initiation and growth. *In vivo*, PRL over-expressing transgenic mice have an increased incidence of mammary tumors [1], while PRL knock-out mice have a reduced incidence of mammary tumors [2]. In women, elevated PRL is associated with increased risk of ER-positive breast cancer [3,4]. Furthermore, 70-95% of human breast cancers express PRL receptor (PRLR) [5,6], and many breast cancer cell lines express high levels of PRLR with evidence of proliferative or survival responses to PRL *in vitro*[7,8]. PRL was also able to enhance 17β-Estradiol (E2) dependent proliferation of breast cancer cells [9-11]. On the other hand, evidence suggests that PRL acts to preserve cellular differentiation of breast cancer. Stat5 transcription factors, principal mediators of PRL effects [12,13], are frequently inactivated during metastatic progression in clinical breast cancer specimens, and loss of Stat5 signaling is associated with unfavorable prognosis and increased risk of anti-estrogen therapy failure [14-17]. In experimental breast cancer models, activation of Stat5 increased cell surface E-cadherin expression, induced homotypic cell clustering, and reduced invasion through Matrigel [18,19]. Restoration of PRL-Stat5 signaling in the mesenchymal-like MDA-MB-231 cells reverted their invasive phenotype, while blocking autocrine PRL signaling in the epithelial T47D cell line was associated with EMT and enhanced invasive properties [20]. In T47D cells, PRL also blocked progestin-induction of a tumor-initiating CK5-positive cell population through a mechanism that involved PRL-suppression of progestin-induced BCL6 [21].

Due to the importance of PRL in breast cancer growth and differentiation, identifying genes regulated by the PRL-Stat5 pathway may provide new insights into the pleiotropic effects of PRL in breast cancer. Several studies have identified genes regulated by PRL in the normal mouse mammary gland [22-26], but only a limited number of studies have been carried out in human breast cancer cells. More importantly, global profiling for PRL-modulated gene expression in human breast cancer *in vivo* has not been reported. One *in vitro* study identified genes regulated by PRL, E2, and PRL + E2 in cultured ER-positive T47D cells using genome-wide transcript profiling [9], while a second *in vitro* study used subtractive hybridization to identify PRL-regulated genes in the ER-negative, Her2-overexpressing SKBR3 cell line [27]. However, neither study confirmed whether the identified transcripts were regulated by Stat5 or remained PRL-modulated *in vivo*. Work from our laboratory used selective overexpression of either Stat5a or Stat5b in the ER-positive MCF-7 human breast cancer cell line *in vitro* followed by PRL exposure to explore differences in Stat5a and Stat5b regulated transcripts on the Affymetrix platform, but this study also failed to confirm any of the modulated transcripts by qRT-PCR or validate the data *in vivo*[17]. An additional very recent study also examined the MCF-7 model and reported PRL regulated genes, as well as genes uniquely modulated through activation of the PRLR transactivation domain [28], but the investigators did not examine whether the identified transcripts were regulated by Stat5 or remained PRL-modulated *in vivo*.

Based on observed differences in hormone-modulated transcriptional programs in human cancer cells *in vitro* and *in vivo*[29], PRL-modified transcripts identified in an *in vivo* environment are expected to be more clinically relevant than transcripts modulated in cells cultured on plastic. The present study reports a panel of 130 PRL regulated transcripts in the human T47D breast cancer xenotransplant model in estrogenized nude mice. T47D xenografts were established in nude mice and mice were exposed to human PRL or saline for 48 h before RNA isolation from tumor extracts. Of the 130 transcripts, 75 were up-regulated and 55 were down-regulated. Modulated transcripts were identified based on fold change (>1.6) and P-value (<0.05). From this initial transcript set, 18 transcripts were selected based on known breast cancer relevance for validation by qRT-PCR. Many but not all of the *in vivo* validated transcripts were PRL-modulated *in vitro*. We also determined the dependence of PRL-modulated transcripts on Stat5, Jak1 or Jak2, and whether individual modulated genes are co-regulated by PRL and E2. One of the PRL-modulated genes identified, parathyroid hormone-related peptide (PTHrP), was found by fluorescence-based quantitative immunohistochemistry to positively correlate with levels of nuclear localized, tyrosine phosphorylated Stat5 (Nuc-pYStat5) at the protein level in clinical human breast cancer specimens. Gene Ontology (GO) analysis of PRL-upregulated genes demonstrated enrichment in differentiation pathways. Finally, a gene signature based on PRL-upregulated genes was associated with prolonged relapse-free and metastasis-free survival in human breast cancer patients. Studies are ongoing to determine how modulation of these genes, including PTHrP, may mediate PRL effects in breast cancer.

## Results

### Global transcript profiling reveals novel PRL-modulated genes in human T47D breast cancer xenotransplants

**Figure 1:**
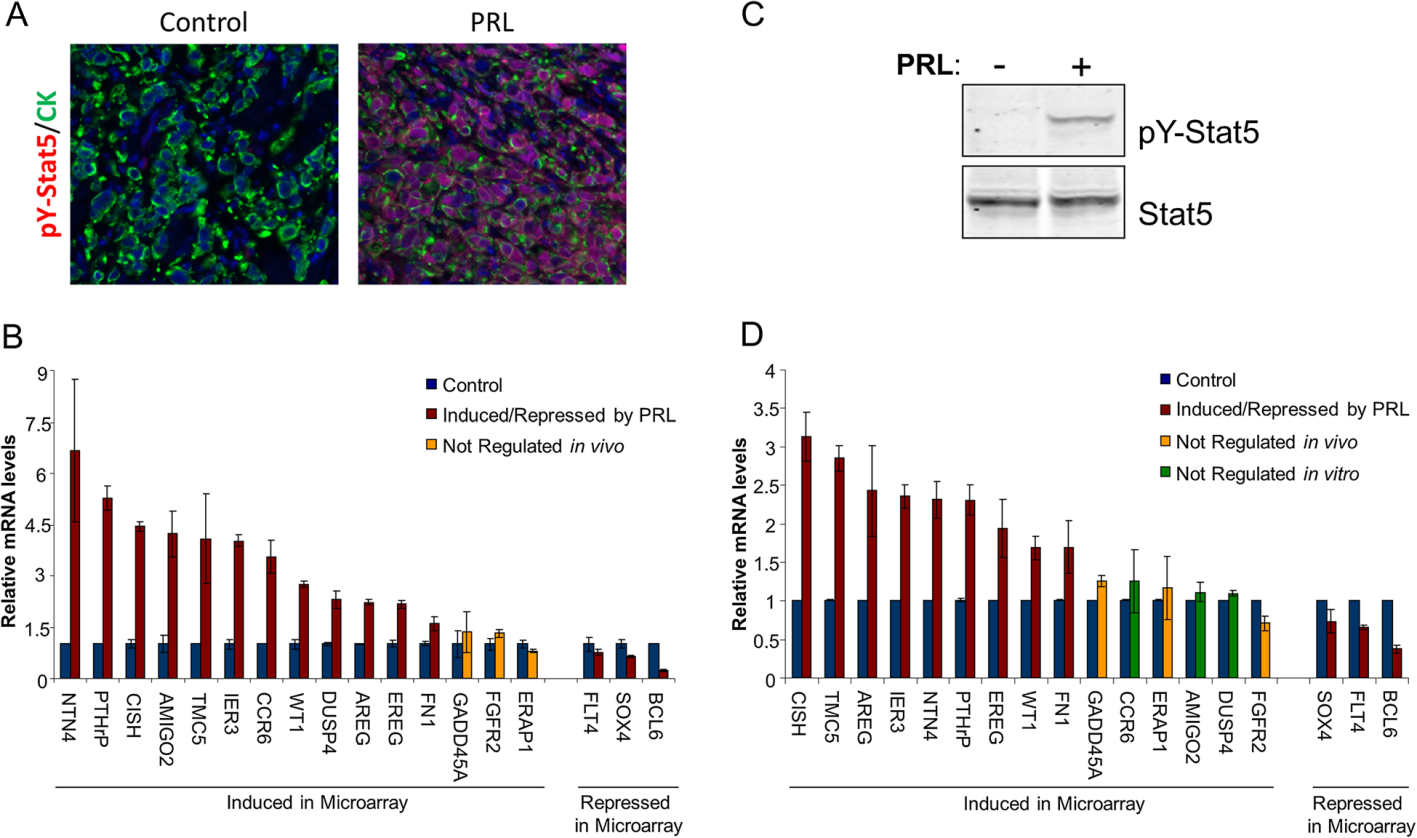
**qRT-PCR validation of 18 candidate prolactin modulated transcripts.** (**A**) Representative images of T47D xenografts treated with or without PRL for 48 h and stained with tyrosine phosphorylated Stat5 (pY-Stat5)(red) or DAPI (blue) using immunofluorescence. (**B**) RNA isolated from T47D xenografts were tested for 18 breast cancer relevant genes using qRT-PCR analysis. Bars represent an average of fold change from 3 independent xenograft RNA samples. (**C**) Immunoblot of pY-Stat5 and Stat5 in T47D cells grown *in vitro* and placed in serum starvation media for 24 h before treatment with vehicle or 10 nM PRL for 24 h. (**D**) RNA isolated from T47D cells grown *in vitro* were tested for the same 18 breast cancer relevant genes using qRT-PCR analysis. Bars represent an average of fold change from 3 independent experiments.

Global gene expression analysis was performed using the hormone receptor positive T47D xenotransplant model. Tumor-bearing nude mice received either human prolactin (N = 10) or vehicle (N = 10) subcutaneously every 12 h for 48 h. Representative images of T47D xenograft tissues display robust tyrosine phosphorylated Stat5 (pY-Stat5) staining in response to PRL (Figure 1A). For the microarray analyses, RNA isolated from individual tumors was pooled into 3 groups from PRL injected mice and 3 groups from control mice, with each group containing RNA from tumors of 3 or 4 mice. Global profiling on the Affymetrix U133 platform identified 75 upregulated transcripts (Table 1) and 55 down-regulated transcripts (Table 2) based on P-values (<0.05) and fold difference (>1.6). From this panel, 18 transcripts were selected for further analysis based on established breast cancer relevance (Table 3). CISH was induced by PRL and included in subsequent analyses as a positive control, since CISH is an established STAT5 target gene [30]. Based on the microarray data of this panel of 18 transcripts, 15 were upregulated and three were down-regulated 1.6-fold.

**Table 1:** Upregulated genes from microarray with fold change >1.6 and p < 0.05

	**Unigene**	**Symbol**	**Gene descriptor**	**Fold change**
1	Hs.89626	PTHrP	parathyroid hormone-like hormone	11.9
2	Hs.473539	BACH1	BTB and CNC homology 1, basic leucine zipper transcription factor 1	5.2
3	Hs.46468	CCR6	chemokine (C-C motif) receptor 6	4.9
4	Hs.314676	ITCH	itchy homolog E3 ubiquitin protein ligase	4.2
5	Hs.150744	INVS	inversin	3.8
6	Hs.387222	NEK6	NIMA (never in mitosis gene a)-related kinase 6	3.8
7	Hs.76095	IER3	immediate early response 3	3.4
8	Hs.121520	AMIGO2	amphoterin induced gene 2	3.3
9	Hs.115263	EREG	epiregulin	3.2
10	Hs.436186	ERAP1	type 1 tumor necrosis factor receptor shedding aminopeptidase regulator	3.0
11	Hs.99037	CTEN	C-terminal tensin-like	3.0
12	Hs.252855	MFI2	antigen p97 (melanoma associated)	2.9
13	Hs.439658	MGC4796	Ser/Thr-like kinase	2.8
14	Hs.417962	DUSP4	dual specificity phosphatase 4	2.7
15	Hs.8257	CISH	cytokine inducible SH2-containing protein	2.6
16	Hs.660427	PAR5	Prader-Willi/Angelman syndrome-5	2.6
17	Hs.279887	AIPL1	aryl hydrocarbon receptor interacting protein-like 1	2.6
18	Hs.145807	TMC5	transmembrane channel-like 5	2.5
19	Hs.512708	TGM2	transglutaminase 2	2.4
20	Hs.270833	AREG	amphiregulin (schwannoma-derived growth factor)	2.4
21	Hs.170623	FGD6	FYVE, RhoGEF and PH domain containing 6	2.3
22	Hs.418138	FN1	fibronectin 1	2.3
23	Hs.102541	NTN4	netrin 4	2.2
24	Hs.354906	RAB39	RAB39, member RAS oncogene family	2.2
25	Hs.443906	EGLN3	egl nine homolog 3 (C. elegans)	2.2
26	Hs.149156	GLDC	glycine dehydrogenase	2.2
27	Hs.1145	WT1	Wilms tumor 1	2.1
28	Hs.25220	LARGE	like-glycosyltransferase	2.1
29	Hs.282557	CP	ceruloplasmin (ferroxidase)	2.0
30	Hs.78909	ZFP36L2	zinc finger protein 36, C3H type-like 2	2.0
31	Hs.413297	RGS16	regulator of G-protein signalling 16	2.0
32	Hs.182454	NYREN18	NEDD8 ultimate buster-1	2.0
33	Hs.96125	RCP	Rab coupling protein	2.0
34	Hs.308028	TMEM17	transmembrane protein 17	2.0
35	Hs.21894	PPM1H	protein phosphatase 1H (PP2C domain containing)	2.0
36	Hs.240395	KCNK6	potassium channel, subfamily K, member 6	1.9
37	Hs.36563	B7-H4	immune costimulatory protein B7-H4	1.9
38	Hs.27345	RNGTT	RNA guanylyltransferase and 5’-phosphatase	1.9
39	Hs.144287	HEY2	hairy/enhancer-of-split related with YRPW motif 2	1.8
40	Hs.269857	HRB2	HIV-1 rev binding protein 2	1.8
41	Hs.387871	TNFSF10	tumor necrosis factor (ligand) superfamily, member 10	1.8
42	Hs.80409	GADD45A	growth arrest and DNA-damage-inducible, alpha	1.8
43	Hs.134742	FAM20C	family with sequence similarity 20, member C	1.8
44	Hs.274701	TK2	thymidine kinase 2, mitochondrial	1.8
45	Hs.55610	SLC30A1	solute carrier family 30 (zinc transporter), member 1	1.8
46	Hs.82173	TIEG	TGFB inducible early growth response	1.8
47	Hs.404081	FGFR2	fibroblast growth factor receptor 2	1.8
48	Hs.31218	SCAMP1	secretory carrier membrane protein 1	1.8
49	Hs.350470	TFF1	trefoil factor 1	1.8
50	Hs.95655	SECTM1	secreted and transmembrane 1	1.8
51	Hs.902	NF2	neurofibromin 2	1.7
52	Hs.310640	T2BP	TRAF2 binding protein	1.7
53	Hs.252550	TNIK	TRAF2 and NCK interacting kinase	1.7
54	Hs.6838	ARHE	ras homolog gene family, member E	1.7
55	Hs.418062	B3GALT3	betaGlcNAc beta 1,3-galactosyltransferase, polypeptide 3	1.7
56	Hs.270411	PLEKHC1	pleckstrin homology domain containing, family C member 1	1.7
57	Hs.647388	ARHGDIG	Rho GDP dissociation inhibitor (GDI) gamma	1.7
58	Hs.202453	MYC	v-myc myelocytomatosis viral oncogene homolog (avian)	1.6
59	Hs.110488	CHSY1	carbohydrate (chondroitin) synthase 1	1.6
60	Hs.9795	ACOX2	acyl-Coenzyme A oxidase 2, branched chain	1.6
61	Hs.158357	UNC5CL	unc-5 homolog C (C. elegans)-like	1.6
62	Hs.441972	IFNT1	interferon tau-1	1.6
63	Hs.221889	CSDA	cold shock domain protein A	1.6
64	Hs.333503	RNF38	ring finger protein 38	1.6
65	Hs.203581	DDX54	DEAD (Asp-Glu-Ala-Asp) box polypeptide 54	1.6
66	Hs.345226	ZNF563	zinc finger protein 563	1.6
67	Hs.30991	ANKRD6	ankyrin repeat domain 6	1.6
68	Hs.4113	AHCYL1	S-adenosylhomocysteine hydrolase-like 1	1.6
69	Hs.416077	SEMA4B	sema domain	1.6
70	Hs.7378	PHLDB2	pleckstrin homology-like domain, family B, member 2	1.6
71	Hs.369063	ZIC2	Zic family member 2 (odd-paired homolog, Drosophila)	1.6
72	Hs.515284	ZNF505	zinc finger protein 505	1.6
73	Hs.426511	MIPOL1	mirror-image polydactyly 1	1.6
74	Hs.108966	PIP5K2A	phosphatidylinositol-4-phosphate 5-kinase, type II, alpha	1.6
75	Hs.432607	PSMB2	proteasome (prosome, macropain) subunit, beta type, 2	1.6

**Table 2:** Downregulated genes from microarray with fold change > −1.6 and p < 0.05

	**Unigene**	**Symbol**	**Gene descriptor**	**Fold Change**
1	Hs.275464	KLK10	kallikrein 10	−3.3
2	Hs.307030	KRTAP1-5	keratin associated protein 1-5	−3.3
3	Hs.282233	MLLT6	myeloid/lymphoid leukemia translocated to, 6	−3.2
4	Hs.78518	NPR2	natriuretic peptide receptor B/guanylate cyclase B	−2.8
5	Hs.155024	BCL6	B-cell CLL/lymphoma 6	−2.8
6	Hs.90800	MMP16	matrix metalloproteinase 16 (membrane-inserted)	−2.5
7	Hs.87539	ALDH3B2	aldehyde dehydrogenase 3 family, member B2	−2.4
8	Hs.144906	METAP2	methionyl aminopeptidase 2	−2.3
9	Hs.440455	ALAS2	aminolevulinate, delta-, synthase 2	−2.2
10	Hs.266175	PAG	phosphoprotein associated with glycosphingolipid-enriched microdomains	−2.2
11	Hs.435947	RBM15	RNA binding motif protein 15	−2.2
12	Hs.233325	HFE	hemochromatosis	−2.1
13	Hs.443012	SEMA6A	sema domain,transmembrane domain(TM),and cytoplasmic domain,(semaphorin)6A	−2.0
14	Hs.357901	SOX4	SRY (sex determining region Y)-box 4	−2.0
15	Hs.415048	FLT4	fms-related tyrosine kinase 4	−2.0
16	Hs.380833	IGFBP5	insulin-like growth factor binding protein 5	−1.9
17	Hs.444881	CRAMP1L	Crm, cramped-like (Drosophila)	−1.9
18	Hs.398124	DNAH5	dynein, axonemal, heavy polypeptide 5	−1.9
19	Hs.79025	SNRK	SNF-1 related kinase	−1.9
20	Hs.432121	PRDX2	peroxiredoxin 2	−1.9
21	Hs.22370	NEXN	nexilin (F actin binding protein)	−1.9
22	Hs.144914	GNMT	glycine N-methyltransferase	−1.9
23	Hs.21446	CENTB5	centaurin, beta 5	−1.9
24	Hs.58103	AKAP9	A kinase (PRKA) anchor protein (yotiao) 9	−1.8
25	Hs.387385	SMURF2	E3 ubiquitin ligase SMURF2	−1.8
26	Hs.324470	ADD3	adducin 3 (gamma)	−1.8
27	Hs.348387	GSTM4	glutathione S-transferase M4	−1.8
28	Hs.58419	TARSH	target of Nesh-SH3	−1.7
29	Hs.174051	SNRP70	small nuclear ribonucleoprotein 70 kDa polypeptide (RNP antigen)	−1.7
30	Hs.211601	MAP3K12	mitogen-activated protein kinase kinase kinase 12	−1.7
31	Hs.23964	SAP18	sin3-associated polypeptide, 18 kDa	−1.7
32	Hs.380929	LDHD	lactate dehydrogenase D	−1.7
33	Hs.390568	ZNF585A	zinc finger protein 585A	−1.7
34	Hs.241305	TRIM16	tripartite motif-containing 16	−1.7
35	Hs.403933	FBXO32	F-box only protein 32	−1.7
36	Hs.434756	AP2E	adaptor-related protein complex 2, epsilon subunit	−1.7
37	Hs.173894	CSF1	colony stimulating factor 1 (macrophage)	−1.6
38	Hs.104555	NPFF	neuropeptide FF-amide peptide precursor	−1.6
39	Hs.307015	KRTAP4-14	keratin associated protein 4-14	−1.6
40	Hs.301961	GSTM1	glutathione S-transferase M1	−1.6
41	Hs.307915	ABCC4	ATP-binding cassette, sub-family C (CFTR/MRP), member 4	−1.6
42	Hs.512000	GP1BB	glycoprotein Ib (platelet), beta polypeptide	−1.6
43	Hs.446297	ZNF498	zinc finger protein 498	−1.6
44	Hs.120396	FRMD4	FERM domain containing 4	−1.6
45	Hs.222901	GRIK4	glutamate receptor, ionotropic, kainate 4	−1.6
46	Hs.464896	ZNF397	zinc finger protein 397	−1.6
47	Hs.16232	CNKSR1	connector enhancer of kinase suppressor of Ras 1	−1.6
48	Hs.255526	DTNA	dystrobrevin, alpha	−1.6
49	Hs.107203	PLAC2	placenta-specific 2	−1.6
50	Hs.9029	KRT23	keratin 23 (histone deacetylase inducible)	−1.6
51	Hs.91753	SMPD3	sphingomyelin phosphodiesterase 3, neutral membrane (neutral sphingomyelinase II)	−1.6
52	Hs.331555	SPINK5	serine protease inhibitor, Kazal type 5	−1.6
53	Hs.391858	TIA1	TIA1 cytotoxic granule-associated RNA binding protein	−1.6
54	Hs.109122	MPP5	membrane protein, palmitoylated 5 (MAGUK p55 subfamily member 5)	−1.6
55	Hs.445072	ARGBP2	Arg/Abl-interacting protein ArgBP2	−1.6

**Table 3:** 18 genes chosen for further study based on breast cancer relevance

	**Unigene**	**Symbol**	**Gene descriptor**	**Fold Change**
1	Hs.89626	PTHrP	parathyroid hormone-like hormone	11.9
2	Hs.46468	CCR6	chemokine (C-C motif) receptor 6	4.9
3	Hs.76095	IER3	immediate early response 3	3.4
4	Hs.121520	AMIGO2	amphoterin induced gene 2	3.3
5	Hs.115263	EREG	epiregulin	3.2
6	Hs.436186	ERAP1	type 1 tumor necrosis factor receptor shedding aminopeptidase regulator	3.0
7	Hs.417962	DUSP4	dual specificity phosphatase 4	2.7
8	Hs.8257	CISH	cytokine inducible SH2-containing protein	2.6
9	Hs.145807	TMC5	transmembrane channel-like 5	2.5
10	Hs.270833	AREG	amphiregulin (schwannoma-derived growth factor)	2.4
11	Hs.418138	FN1	fibronectin 1	2.3
12	Hs.102541	NTN4	netrin 4	2.2
13	Hs.1145	WT1	Wilms tumor 1	2.1
14	Hs.80409	GADD45A	growth arrest and DNA-damage-inducible, alpha	1.8
15	Hs.404081	FGFR2	fibroblast growth factor receptor 2	1.8
16	Hs.155024	BCL6	B-cell CLL/lymphoma 6	−2.8
17	Hs.357901	SOX4	SRY (sex determining region Y)-box 4	−2.0
18	Hs.415048	FLT4	fms-related tyrosine kinase 4	−2.0

### qRT-PCR validation of 18 candidate prolactin modulated genes

To validate the data from the global microarray analysis, qRT-PCR analysis was carried out on the 18 selected genes using the same xenograft RNA samples that were used for the microarray-based profiling. Of the upregulated transcripts, 12 out of 15 transcripts were confirmed to be upregulated by qRT-PCR >1.6-fold, whereas upregulation of GADD45A, ERAP1, FGFR2 was not confirmed by qRT-PCR. Among the selected three down-regulated transcripts, BCL6 was confirmed down-regulated >1.6 fold by qRT-PCR, whereas FLT4 and SOX4 were down-regulated 1.3-fold and 1.6-fold, respectively, by qRT-PCR analysis (Figure 1B).

We then identified PRL-modulated transcripts in T47D xenotransplants that were also modulated by PRL T47D cells *in vitro*. Immunoblot analysis of T47D cells cultured *in vitro* displayed robust pY-Stat5 in response to 10 nM human PRL for 24 h (Figure 1C). qRT-PCR analysis of mRNA extracted from T47D cells treated with or without PRL for 24 h established that 9 of the 12 qRT-PCR validated upregulated transcripts also were upregulated over 1.6-fold *in vitro*, while three transcripts, CCR6, AMIGO2, and DUSP4 were not modulated by PRL *in vitro* (Figure 1D). Transcripts for GADD45A, ERAP1, FGFR2, which were not confirmed by qRT-PCR analysis of transcripts in vivo, remained unmodulated *in vitro* (Figure 1D). Out of the three selected down-regulated transcripts, BCL6 maintained PRL induced down-regulation of >1.6-fold *in vitro*, whereas FLT4 and SOX4 again were reproducibly down-regulated but only 1.5-fold and 1.4-fold respectively (p < 0.05) (Figure 1D). Since these values were only marginally lower than 1.6, we elected to keep FLT4 and SOX4 in the subsequent analyses. In subsequent experiments we focused on the set of 12 transcripts out of original selected panel of 18 transcripts that were PRL-responsive both *in vivo* and *in vitro*.

### Stat5 regulates novel PRL-modulated transcripts

**Figure 2:**
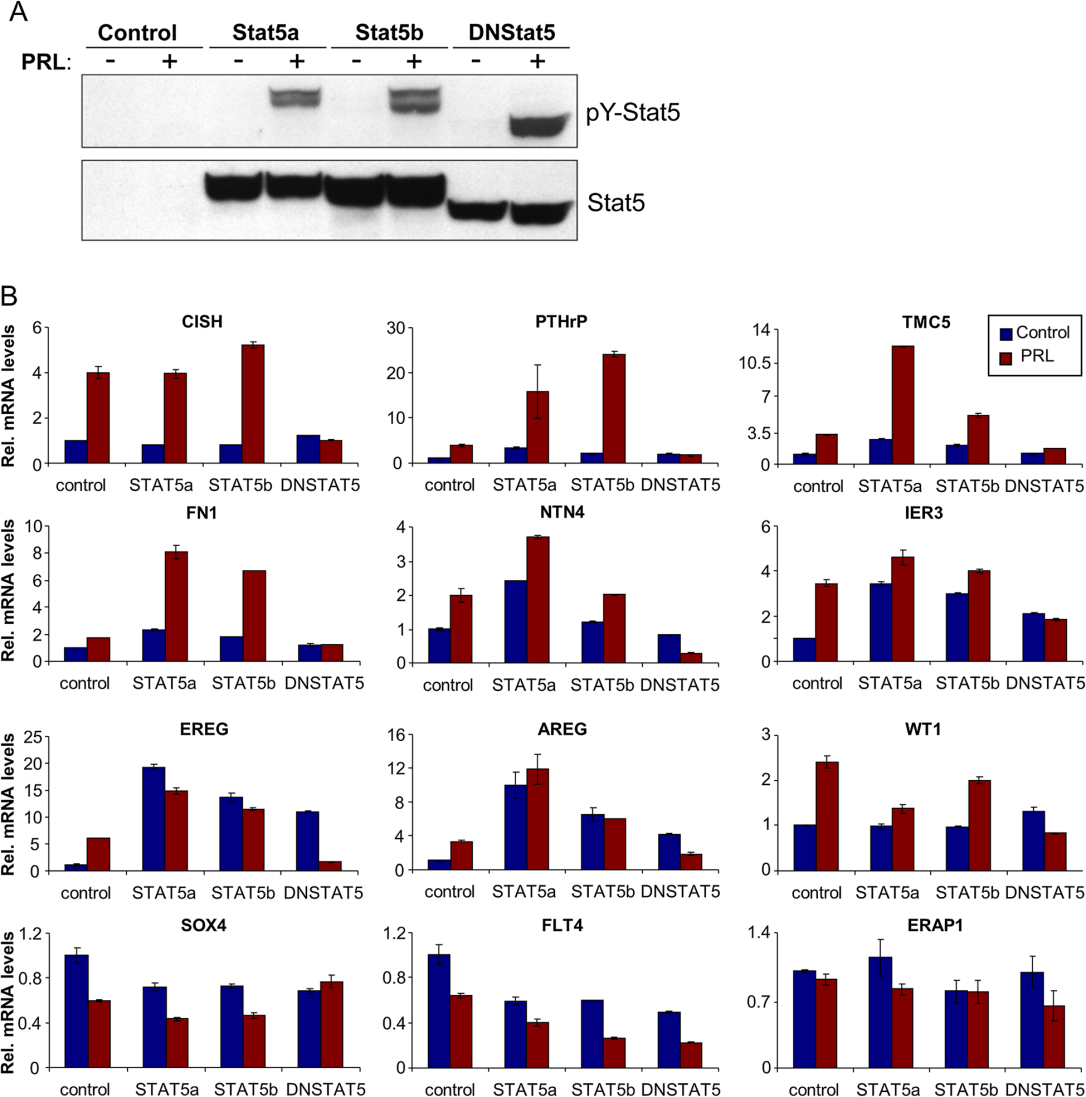
**Stat5 regulates novel prolactin modulated transcripts.** (**A**) Immunoblot of pY-Stat5 and Stat5 in cells treated with adenovirus containing LacZ (Control), Stat5a, Stat5b, or Dominant-Negative Stat5 (DNStat5) for 24 h, then serum starved and treated with vehicle or 10 nM PRL for 24 h. (**B**) RNA isolated from T47D cells treated with adenovirus was tested for 12 PRL modulated transcripts using qRT-PCR analysis.

PRL activates multiple signaling pathways in breast cancer cells [31], with Stat5 constituting a principal mediator of PRL actions during development and differentiation of the mammary gland [32]. To determine whether the observed PRL-modulated transcripts were regulated through the Stat5 pathway, we overexpressed Stat5a, Stat5b, or a dominant-negative Stat5a/b (DNStat5) in T47D cells using adenoviral gene delivery, and treated cells with or without PRL for 24 h. Immunoblot analysis of total cell lysates verified over-expression of the correct Stat5 variants and robust PRL-induction of pY-Stat5 in cells following adenoviral gene delivery (Figure 2A). The overexpression was sufficiently high that basal levels of Stat5 were not detectable without further exposure of the blots (not shown). qRT-PCR analysis revealed that DNStat5 blocked PRL induction of each of the 9 upregulated transcripts. PRL induction of most of these 9 transcripts were further enhanced by overexpression of Stat5a or Stat5b, either basal or PRL-induced (Figure 2B). Although levels of PRL-induced CISH and WT1 transcripts did not rise further by Stat5 overexpression, PRL-induction of both transcripts was effectively abrogated by DNStat5, thus supporting Stat5-mediated regulation of these genes. Stat5 overexpression also enhanced down-regulation of both SOX4 and FLT4. The ERAP1 transcript was included as a negative control since it was not regulated by PRL as shown in Figure 1. Stat5a, Stat5b, or DN-Stat5 overexpression had no effect on ERAP1 transcript levels, indicating that enhancement of PRL-induction detected for the other transcripts is specific (Figure 2). BCL6 was omitted from this analysis since we have published this separately [33].

### Jak2 and Jak1 are critical for PRL gene regulation in T47D breast cancer cells

**Figure 3:**
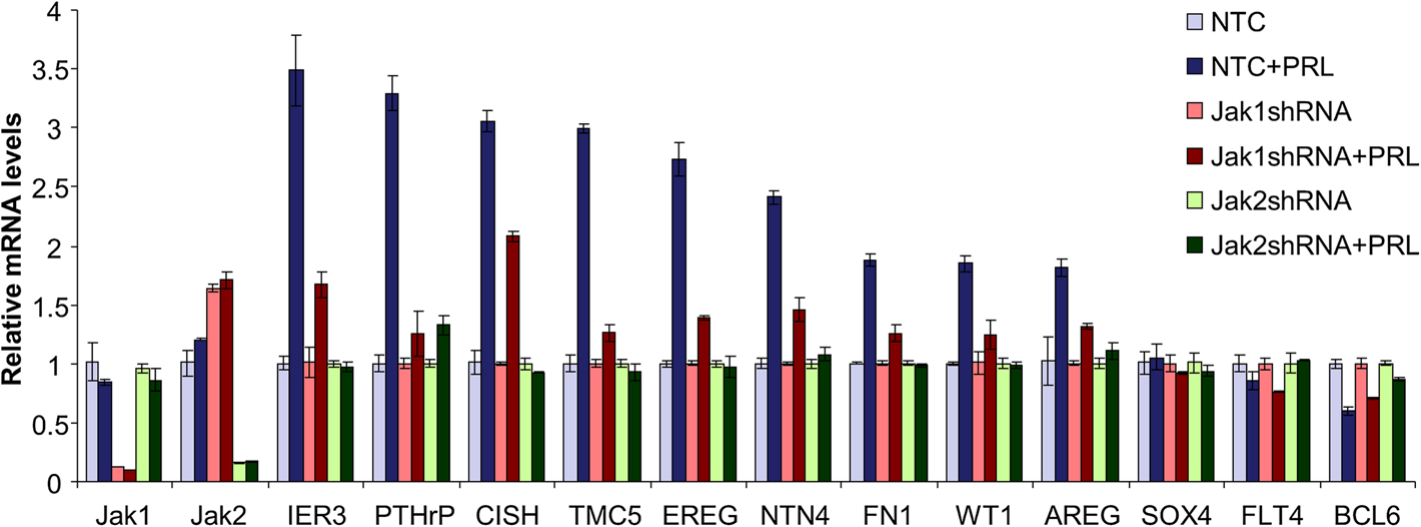
**Jak2 and Jak1 are critical for PRL transcript regulation in T47D breast cancer cells.** T47D cells were treated with lentivirus containing NTC, Jak1 shRNA, or Jak2 shRNA overnight. Then, cells were placed in serum starvation media for 24 h and then treated with vehicle or 10 nM PRL for another 24 h before RNA isolation and qRT-PCR analysis of the 12 PRL regulated transcripts.

Conditional gene knock-out in mice demonstrated that Jak2 is the key Stat5 tyrosine kinase in breast epithelial cells during and outside of pregnancy and lactation [34]. However, we have reported that in human breast cancer cell lines Jak1 is also recruited in a Jak2-dependent manner for maximal PRL-activation of Stat5 and other signaling mediators [31]. To determine whether PRL-recruitment of Jak1 was required for maximal modulation of PRL-regulated transcripts, T47D cells were infected with lentivirus carrying shRNAs targeting either Jak1 or Jak2 followed by treatment with or without PRL for 24 h. Jak1 shRNA was effective and selective, as judged by marked down-regulation of Jak1 mRNA but not Jak2 mRNA (Figure 3). Conversely, Jak2 shRNA effectively suppressed Jak2 mRNA but not Jak1 mRNA (Figure 3). All nine PRL-upregulated transcripts showed complete dependence on Jak2 (Figure 3). Importantly, all nine PRL-induced transcripts were also partially suppressed by Jak1 knockdown, consistent with a significant role for Jak1 recruitment by PRL in breast cancer cells to maximize downstream signals. Among the three down-regulated transcripts, BCL6 down-regulation by PRL was dependent on Jak2 but not on Jak1, whereas down-regulation of FLT4 and SOX4 by PRL was not significant under these experimental conditions, possibly due to cell stress during lentiviral infection (Figure 3). Collectively, these data are consistent with a general model in which Jak2 is critical for PRL receptor signaling and Jak1 recruitment is needed for maximal signal.

### Synergistic enhancement by E2 of PRL-induced proliferation and select PRL-modulated genes

**Figure 4:**
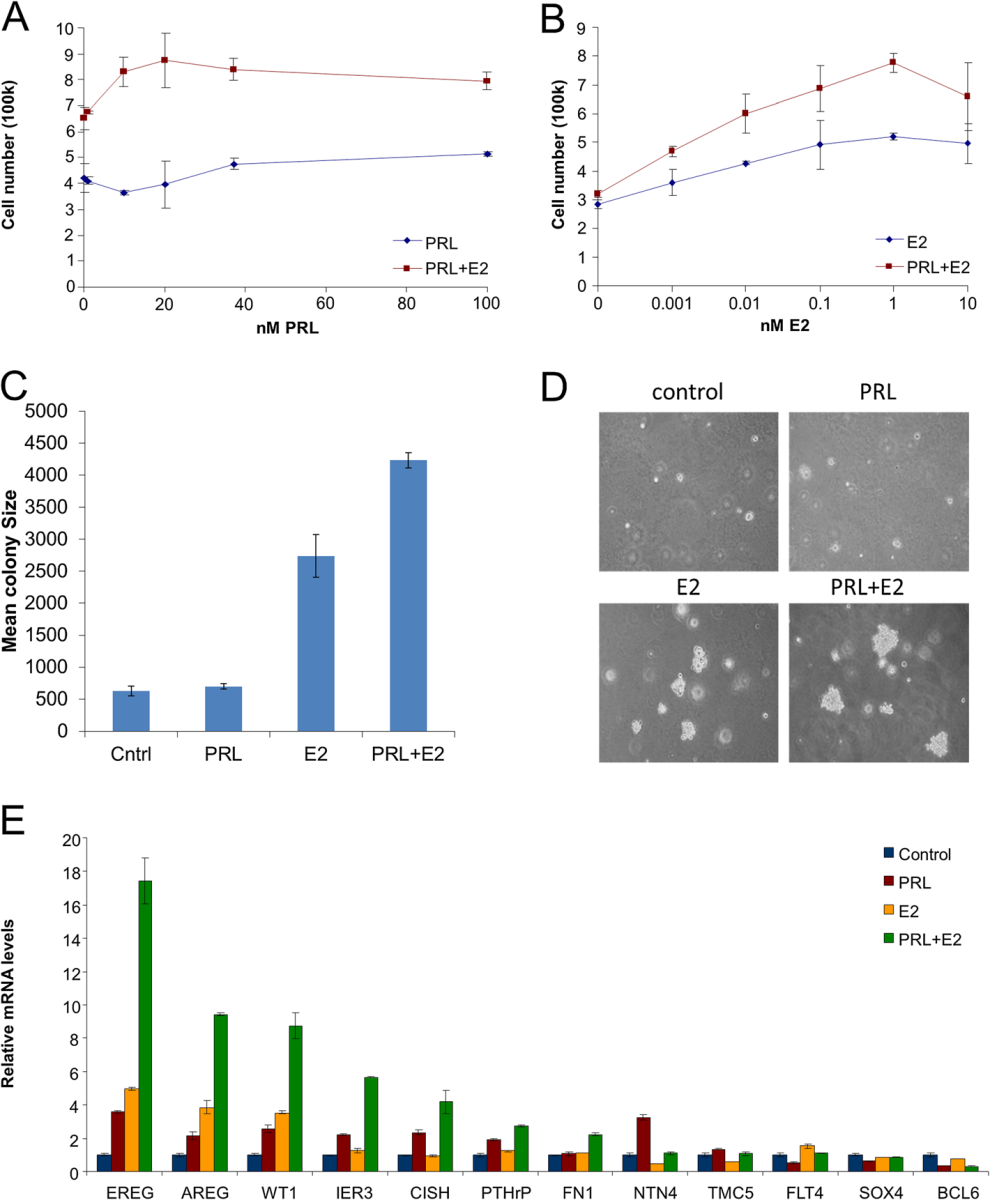
**Enhancement by E2 of PRL-induced proliferation and select PRL modulated transcripts.** (**A**) T47D cells were treated with varying doses of PRL (0, 1, 10, 20, 37, and 100 nM) in the presence of constant E2 (1 nM) for 72 h. Bars represent triplicates of each condition that were counted and averaged. (**B**) T47D cells were treated with varying doses of E2 (0.001, 0.01, 0.1, 1, 10 nM) in the presence of constant PRL (20 nM) for 72 h. Bars represent triplicates of each condition that were counted and averaged. (**C**) T47D cells were treated with vehicle, 20 nM PRL, 1 nM E2, or 20 nM PRL + 1 nM E2 for 2 weeks. Images from four independent wells were analyzed for colony size through ImageJ. (**D**) Representative images of T47D cells grown on soft agar and treated with vehicle, PRL, E2, or PRL + E2 for 2 weeks. (**E**) T47D cells were treated with PRL, E2, or PRL + E2 for 24 h. RNA was isolated and qRT-PCR analysis was performed on the 12 PRL modulated transcripts.

Previous studies have indicated that PRL may enhance E2-induced proliferation as well as additively or synergistically increase transcription of certain PRL or E2 target genes [9-11]. To verify that the PRL-E2 interaction occurred in T47D cells under our culture conditions, we treated T47D cells with varying doses of PRL (0, 1, 10, 20, 37, and 100 nM) while keeping E2 constant (1 nM), and determined cell number after 72 h. PRL concentrations as low as 10 nM in the presence of E2 were associated with an increase in cell number compared to E2 alone, and this effect was maintained at higher PRL concentrations (Figure 4A). Next, we maintained constant PRL concentration (20 nM) while varying the E2 dose (0.001, 0.01, 0.1, 1, 10 nM), and counted cell numbers after 72 h. At every concentration of E2, we observed a PRL-induced increase in cell number (Figure 4B). We also determined the effect of PRL on E2-driven growth in soft agar and measured colony size after 2 weeks. While PRL alone had limited effect on colony size, E2 increased colony size dramatically, and PRL further increased E2-driven colony size (Figure 4C). Representative images of the colonies in soft agar are displayed in Figure 4D. These data established under our culture conditions that PRL positively interacts with E2 to induce proliferation of luminal T47D breast cancer cells.

To determine whether the PRL-modulated transcript panel was affected by co-treatment with E2, we treated T47D cells with vehicle, PRL, E2, or PRL + E2 for 24 h and performed qRT-PCR analysis of the 12 transcripts. Seven out of the nine upregulated transcripts displayed further induction with E2 present (NTN4 and TMC5 had no further induction) (Figure 4E). None of the three PRL-downregulated genes displayed further downregulation in the presence of E2 (Figure 4E). This data indicates that E2 is selectively modulating some but not all PRL-Stat5 regulated transcripts, and these specific transcripts may be playing a role in PRL’s ability to increase E2-driven breast cancer cell proliferation.

### PTHrP protein levels correlate with levels of pYStat5 in human breast cancer tissues

**Figure 5:**
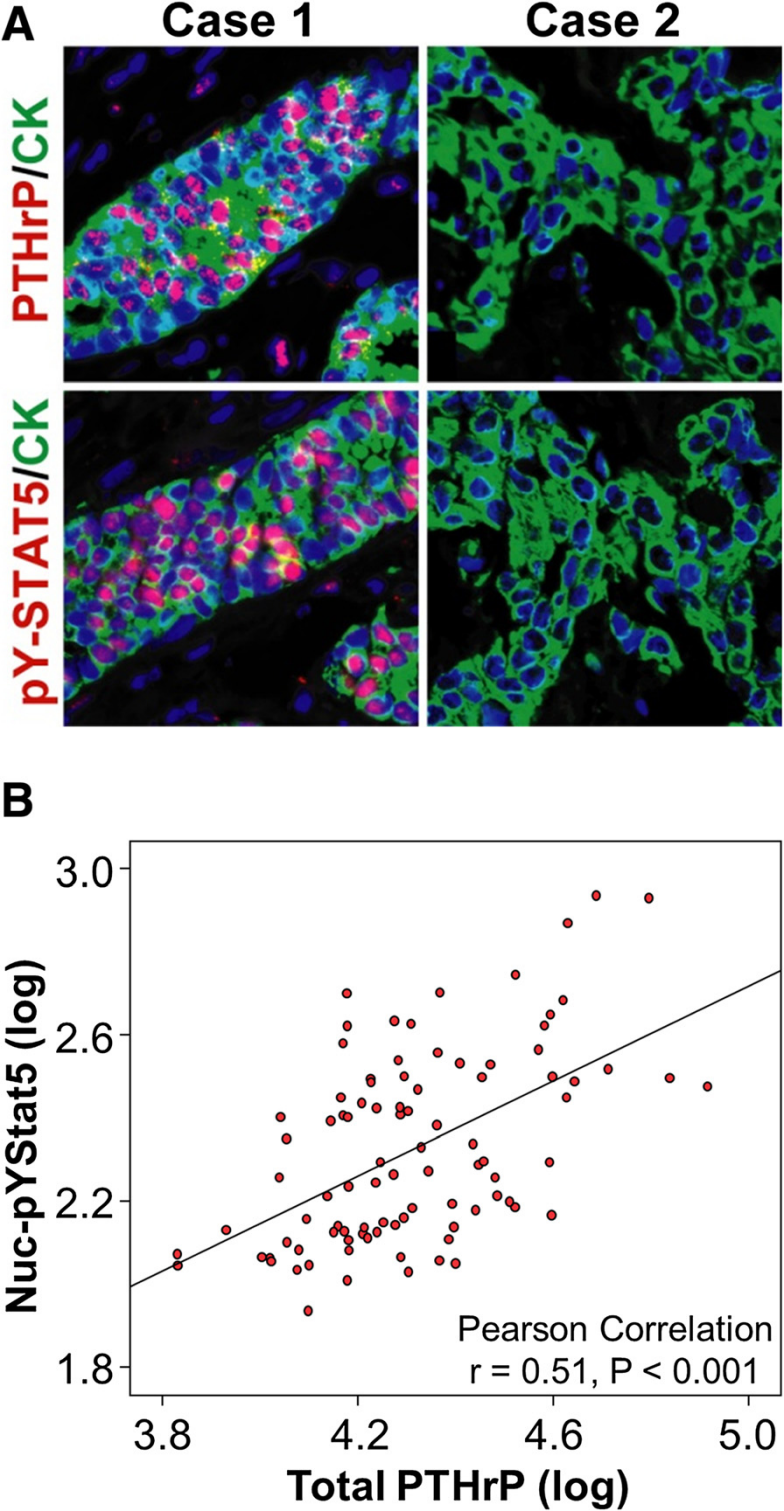
**PTHrP protein levels correlate with levels of nuc-pYStat5 in human breast cancer tissues.** (**A**) Representative immunofluroescent images of two cases of breast cancer stained for pY-Stat5 (Red), PTHrP (Red), cytokeratin (Green), and DNA (Blue) using immunohistochemistry. (**B**) Scatter plot and Pearson correlation analysis between nuc-pYStat5 and cellular PTHrP protein levels on 92 breast cancer specimens.

To begin to assess the clinical relevance of the newly identified PRL-modulated transcripts, we selected the gene product most strongly upregulated by PRL in the *in vivo* xenotransplant tumors, PTHrP, for protein expression analysis in clinical breast cancer specimens. We hypothesized that cellular PTHrP protein levels would be positively correlated with levels of nuclear localized and tyrosine phosphorylated Stat5 (Nuc-pYStat5). Nuc-pYStat5 is an indirect measure of PRL receptor activation in breast epithelia, and we documented evidence of Stat5-dependence of PRL-upregulation of PTHrP in T47D cells. Levels of cellular PTHrP and Nuc-pYStat5 were analyzed using fluorescence-based quantitative immunohistochemistry on a breast cancer progression array containing 40 normal and 140 malignant breast tissues. Representative images of PTHrP and pY-Stat5 staining are shown in Figure 5A, where Case 1 has high PTHrP and Nuc-pYStat5 levels, while Case 2 has low PTHrP and Nuc-pYStat5 levels. Evaluable levels of cellular PTHrP and Nuc-pYStat5 were obtained in 92 breast cancer specimens and in support of our hypothesis, a statistically significant positive correlation was detected (r = 0.51, P < 0.001) (Figure 5B).

### Gene ontology (GO) terms are enriched in differentiation markers

Analysis of GO biological process terms using the 75 PRL-upregulated transcripts identified in this study revealed 24 GO terms that had a false discovery rate (FDR) below 25% (Table 4). Many of the pathways that were identified were homeostasis-related, correlating with the ability of the PRL-Stat5 pathway to maintain cellular differentiation, consistent with established pro-differentiation effects of PRL on normal and malignant luminal breast epithelial cells. In addition, proliferation-regulation and negative regulation of apoptosis were also identified, which is consistent with the reported role of PRL contributing to breast cancer initiation and growth.

**Table 4:** Gene Ontology (GO) analysis of PRL upregulated genes with false discovery rate (FDR) < 25

**GO Term**	**Description**	**P value**	**FDR**
GO:0030005	cellular di-, tri-valent inorganic cation homeostasis	0.002	2.452
GO:0042592	homeostatic process	0.002	2.848
GO:0055066	di-, tri-valent inorganic cation homeostasis	0.002	3.079
GO:0030003	cellular cation homeostasis	0.003	4.015
GO:0055080	cation homeostasis	0.004	6.550
GO:0006879	cellular iron ion homeostasis	0.006	8.210
GO:0043123	positive regulation of I-kappaB kinase/NF-kappaB cascade	0.006	9.136
GO:0060249	anatomical structure homeostasis	0.008	10.970
GO:0055072	iron ion homeostasis	0.008	11.045
GO:0019725	cellular homeostasis	0.008	11.791
GO:0043122	regulation of I-kappaB kinase/NF-kappaB cascade	0.008	11.809
GO:0048514	blood vessel morphogenesis	0.009	12.950
GO:0051052	regulation of DNA metabolic process	0.010	13.580
GO:0042127	regulation of cell proliferation	0.010	13.992
GO:0043066	negative regulation of apoptosis	0.011	15.532
GO:0043069	negative regulation of programmed cell death	0.012	16.371
GO:0060548	negative regulation of cell death	0.012	16.542
GO:0048878	chemical homeostasis	0.013	17.642
GO:0006873	cellular ion homeostasis	0.014	18.854
GO:0055082	cellular chemical homeostasis	0.014	19.975
GO:0001568	blood vessel development	0.015	20.556
GO:0010627	regulation of protein kinase cascade	0.015	20.807
GO:0001944	vasculature development	0.016	22.079

### Prolactin-upregulated gene signature is associated with prolonged time to relapse and metastasis

**Figure 6:**
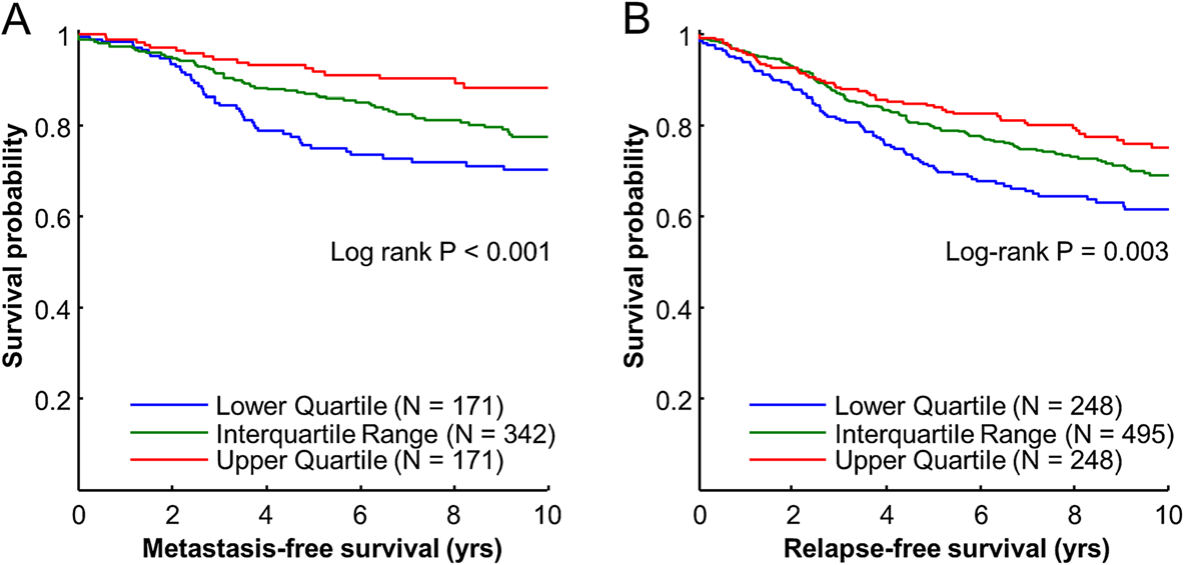
**PRL upregulated gene signature is associated with metastasis-free and relapse-free survival**. (**A**) Patients with high levels of a genes signature of PRL-upregulated had reduced risk of developing metastasis compared to patients with low levels of the PRL-upregulated gene signature. (**B**) Patients with high levels of the PRL-induced gene signature had prolonged relapse-free survival compared to patients with low levels of the PRL gene signature.

We then determined whether the PRL-upregulated gene signature was associated with clinical outcome, using an available 49 of 75 PRL-upregulated genes (Table 5) in a cohort of 936 primary invasive breast cancer patients. We divided the patients into three groups based on their degree of expression of the PRL-gene signature (upper quartile, interquartile range, and lower quartile). The patients in the upper quartile had significantly prolonged time to metastasis compared to patients associated with the lower quartile (Figure 6A). In addition, patients that were in the upper quartile also had significantly prolonged disease-free survival than patients in the lower quartile (Figure 6B).

**Table 5:** PRL-upregulated genes used to generate PRL gene signature

**Gene Symbol**	**Correlation**	**Gene symbol**	**Correlation**
	**with signature average**		**with signature average**
AREG	0.6	TNIK	0.25
DUSP4	0.51	PPM1H	0.17
PTHLH	0.46	PAR5	0.15
EREG	0.45	INVS	0.14
SCAMP1	0.43	CSDA	0.13
TMC5	0.42	MYC	0.12
GADD45A	0.42	WT1	0.09
CHSY1	0.41	CISH	0.08
TFF1	0.4	CP	0.07
FGD6	0.39	RGS16	0.07
BACH1	0.38	NF2	0.07
AHCYL1	0.37	FGFR2	0.04
EGLN3	0.34	RNGTT	0.03
AMIGO2	0.33	FN1	0.02
TNFSF10	0.33	ITCH	−0.01
IER3	0.33	LARGE	−0.11
ANKRD6	0.32	PSMB2	−0.12
ACOX2	0.31	SECTM1	−0.15
SLC30A1	0.31	GLDC	−0.2
ERAP1	0.31	AIPL1	−0.21
RNF38	0.3	TGM2	−0.22
ZFP36L2	0.3	DDX54	−0.23
CCR6	0.28	MFI2	−0.27
HEY2	0.25	ARHGDIG	−0.27
TK2	0.25		

## Discussion

The present study reports a novel panel of PRL-modulated transcripts based on analysis of human breast cancer xenograft tumors *in vivo*. We identified 75 up-regulated and 55 down-regulated transcripts that were significantly modulated based on at least a 1.6-fold change with a P-value less than 0.05. From this panel of 130 PRL-modulated transcripts, a subset of 18 transcripts with established breast cancer-relevance was selected for further analysis and validation. Validation by qRT-PCR documented significant modulation of 12 of 18 transcripts *in vitro*. We further documented that the majority of *in vitro*-modulated transcripts were Stat5- and Jak2-dependent, and showed that Jak1 was required for maximal PRL-modulation. Consistent with PRL-enhancement of E2-driven proliferation of breast cancer cells, select PRL-modulated transcripts displayed positive co-regulation by E2, including the growth factors EREG and AREG. These molecular results are summarized in Table 6. Furthermore, quantitative immunofluorescence analyses of clinical breast cancer specimens from a cohort of 92 patients documented a significant positive correlation between tumor levels of PTHrP protein, one of the PRL-stimulated transcripts, and nuclear localized and tyrosine phosphorylated Stat5, a marker of PRL signaling. Gene ontology analysis revealed that prolactin-upregulated genes were associated most frequently with terms involved in homeostasis and differentiation. Finally, a gene signature generated with PRL-upregulated genes was associated with prolonged relapse-free survival as well as metastasis-free survival in a cohort of breast cancer patients. Collectively, the validation studies support the value of the transcript data and are expected to facilitate better understanding of PRL action in breast cancer.

**Table 6:** Summary of transcript regulation

**Gene**	**Fold Change (microarray)**	***in vivo *Regulation**	***in vitro *Regulation**	**Stat5 Regulation**	**Jak2 Regulation**	**Jak1 Regulation**	**E2 Regulation**
PTHrP	11.9	Y	Y	Y	Y	Y	Y
CCR6	4.9	Y	N	Y	Y	Y	Y
IER3	3.4	Y	Y	Y	Y	Y	Y
AMIGO2	3.3	Y	N	NA	NA	NA	NA
EREG	3.2	Y	Y	Y	Y	Y	Y
ERAP1	3.0	N	N	NA	NA	NA	NA
DUSP4	2.7	Y	N	NA	NA	NA	NA
CISH	2.6	Y	Y	Y	Y	Y	Y
TMC5	2.5	Y	Y	Y	Y	Y	N
AREG	2.4	Y	Y	Y	Y	Y	Y
FN1	2.3	Y	Y	Y	Y	Y	Y
NTN4	2.2	Y	Y	Y	Y	Y	N
WT1	2.1	Y	Y	Y	Y	Y	Y
GADD45A	1.8	N	N	NA	NA	NA	NA
FGFR2	1.8	N	N	NA	NA	NA	NA
BCL6	−2.8	Y	Y	Y	Y	N	N
SOX4	−2.0	Y	Y	Y	NA	NA	N
FLT4	−2.0	Y	Y	Y	NA	NA	N

PRL activates both Stat5a and Stat5b, which have 92% amino acid similarity [35], but are encoded by different genes and may mediate overlapping and distinct effects in breast cancer cells [17,33,36-38]. In the present study, which focused on PRL-modulated transcripts in the T47D breast cancer model, experimental overexpression of Stat5a or Stat5b enhanced to a comparable extent PRL-modulation of most transcripts tested. However, TMC5, NTN4, and AREG displayed greater degree of enhanced PRL-modulation when Stat5a was overexpressed rather than Stat5b, supporting the notion that certain genes are more responsive to Stat5a than Stat5b. PRL-modulation of all examined transcripts with the exception of PRL-induced down-regulation of FLT4 mRNA was disrupted by overexpression of a C-terminally truncated Stat5 variant that acts as a dominant-negative molecule for both Stat5a and Stat5b. This exception is consistent with previous reports that repression of certain Stat5 target genes is unaffected by alterations in the Stat5 transactivation domain [33,39]. We have recently reported that Stat5a but not Stat5b expression was lost during progression of human breast cancer, and in cultured MCF-7 cells there was only a limited overlap in transcripts modulated by the two PRL-activated transcription factors [17]. It is possible that in T47D cells the higher basal levels of Stat5a and especially Stat5b make it more difficult to ascertain differences between the two transcription factors in overexpression studies. Future transcript analyses will focus on further characterizing the differences in gene regulation between Stat5a and Stat5b in human breast cancer.

Jak1 was reported to be activated by PRL signaling in human breast cancer lines and cooperate with Jak2 to enhance signaling pathways downstream of PRL receptors, including Stat5, Stat3 and Erk activation [31]. The present study validates the notion that Jak1-coactivation enhances PRL-Jak2 signaling by demonstrating that maximal PRL-modulation of target transcripts required PRL-induced co-activation of Jak1. Future studies to identify the mechanism of activation of Jak1 by PRL receptors in breast cancer and the effect of Jak1-activation on PRL-modulated biology of breast cancer are now warranted.

Furthermore, whereas PRL alone exerted limited proliferative effect on T47D breast cancer cells *in vitro*, PRL enhanced E2-driven cell proliferation both on plastic and soft agar. PRL positively interacted with E2 to further elevate several transcripts encoding growth and progression factors for breast cancer, including AREG, EREG, PTHrP and WT1. Considering the established role for AREG as a paracrine mediator of E2-induced proliferation of luminal breast epithelial cells during pubertal growth [40], AREG may be directly involved in PRL stimulation of E2-driven growth of human breast cancer. A recent study has implicated PRL receptors in the maintenance of ER expression and responsiveness of breast cancer cells to estrogen [28], which is consistent with our findings which demonstrate significant crosstalk between the two pathways. Since Stat5 has been associated with response to anti-estrogen therapy in clinical breast cancer specimens [16,17], this synergistic stimulation of proliferation with estrogen may be a mechanism behind increased responsiveness to anti-estrogen treatments. In addition, PRL biological action may vary depending on the hormonal environment, especially given our recent observation that PRL effectively counteracts progestin-induction of the tumor-initiating CK5-positive cell population [21].

We identified PRL-suppression of BCL6 transcript and protein based on this global transcript analysis of T47D xenotransplants tumors, and we reported a negative correlation between levels of BCL6 protein and Nuc-pYStat5 in clinical breast cancer specimens [33]. The fact that the protein products of two of the PRL-modulated genes identified in this global transcript profiling, PTHrP and BCL6, both correlated with Nuc-pYStat5 in human clinical breast cancer specimens indicates that transcripts identified in the present study are relevant in clinical specimens and may become useful human breast tumor markers of PRL activation.

Supporting the validity of the identified panel of PRL-modulated transcripts in T47D cells *in vivo*, 12 out of the 57 transcripts identified as PRL-modulated transcripts in a recent *in vitro* transcript profiling study of T47D cells overlapped with our panel (AREG, WT1, PTHrP, IER3, TMC5, CISH, BCL6, DUSP4, TNIK, EGLN3, FBXO32, and AKAP9) [9]. Only WT1 and IER3 transcripts were tested and confirmed by qRT-PCR in the previous report. In addition, select transcripts identified in the previous study such as WT1 and IER3 demonstrated co-regulation by E2 [9], consistent with the findings of the present study. *In vitro* transcript profiling for PRL-modulated transcripts in another ER-positive human breast cancer cell line, MCF7, using Stat5 overexpression to enhance PRL effects, yielded 300 candidate PRL-modulated transcripts among which 12 overlapped with the panel identified in the present study of T47D cells (CISH, EGLN3, KCNK6, PTHrP, FN1, CHSY1, BCL6, DUSP4, IGFBP5, TNIK, ABCC4, and MYC) [17]. The limited overlap between our current study of T47D cells and two recent studies of MCF-7 cells *in vitro* may be due to lower expression levels of Stat5 in MCF-7 cells, making it necessary to overexpress Stat5 [17] or PRL receptor [28]. While previous studies were performed *in vitro* and did not broadly validate identified candidate PRL-modulated transcripts by qRT-PCR, the present study also provides novel data by demonstrating PRL-regulation of transcripts *in vivo* and through Jak-Stat5 dependent mechanisms.

The established breast cancer relevance for AREG, WT1, and IER3 is discussed in a previous transcript profiling study [9]. NTN4 is a transmembrane protein whose expression levels positively correlates with better prognosis in breast cancer [41]. TMC5 is a transmembrane channel that was overexpressed in PIK3CA-mutated breast cancer [42]. FN1 is a protein present in the extracellular matrix that is a candidate serum biomarker for detecting breast cancer [43], and disrupting the interaction between FN1 and integrins in breast cancer cells led to increased apoptosis and response to radiation [44]. EREG is a ligand for EGFR and Her4, and was reported to be part of a set of four genes that promote breast cancer intravasation and metastasis to the lung [45]. PTHrP is a secreted protein critical for mammary gland development [46], and extensive research has been performed on its role in mediating breast cancer metastasis to bone [47-49]. SOX4 is a transcription factor regulated by progesterone in breast cancer cells [50], and was identified as an oncogene in prostate cancer [51]. FLT4 is a member of the VEGF receptor family, and expression in vessels surrounding breast tumors was correlated to lymph node positivity and poor clinical outcome [52]. These genes clearly demonstrate the complexity of PRL effects, since PRL has the ability to suppress oncogenes (SOX4, FLT4, BCL6) and upregulate favorable prognostic markers (NTN4), while also upregulating genes involved in breast cancer growth and progression (AREG, WT1, IER3, EREG, TMC5, FN1, PTHrP). These observations are consistent with the many reported conflicting and likely context-dependent effects of PRL in breast cancer.

Furthermore, gene ontology (GO) analysis based on PRL-upregulated genes demonstrated a concentration in homeostasis pathways, consistent with the known pro-differentiation role of PRL in breast cancer. However, other terms such as cell proliferation and anti-apoptosis were also enriched, most likely reflecting the duality of PRL action. Consistent with the enrichment of differentiation terms in the GO analysis, a PRL gene signature generated on PRL-upregulated genes was associated with prolonged time to relapse and metastasis-free survival. These associations are consistent with the multiple reports that Stat5 is associated with favorable prognosis in breast cancer patients [14-16]. In addition, we have recently reported that prolactin can suppress a therapy-resistant, tumor-initiating CK5-positive population induced by progestin [21]. Our results are consistent with a role of PRL in reducing the tumor-initiating CK5-positive cell population, which is implicated in breast cancer metastasis and relapse.

## Conclusions

The present study is the first to report a panel of PRL-modulated transcripts based on global transcript profiling of human breast cancer xenotransplant tumors *in vivo*. Some but not all transcripts were also modulated by PRL *in vitro*. PRL-enhancement of E2-driven proliferation of T47D cells *in vitro* may be mediated by observed co-regulation by PRL and E2 of growth-promoting genes including AREG, EREG, WT1 and PTHrP. PRL-modulated transcripts reported in this study are expected to facilitate deciphering of the mechanisms underlying the pleotropic effects of PRL on breast cancer. PRL-upregulated genes were frequently associated with differentiation pathways. Finally, select transcripts or their protein complement identified in this study also may be useful as breast cancer tumor marker signatures of PRL activation, which is highly relevant considering our report that PRL gene signature is associated with relapse-free survival and metastasis-free survival, and the already documented association of Stat5 with breast cancer prognosis and hormone therapy responsiveness.

## Materials and methods

### Tissue culture

T47D cells were cultured in RPMI (Cellgro) containing 10% fetal bovine serum (FBS) and 1 mM sodium pyruvate (Cellgro). For PRL induction, confluent T47D cells were put in serum starvation media (RPMI without FBS) for 24 h, and then stimulated with either PBS vehicle or 10 nM of recombinant human prolactin (AFP795, provided by Dr. A.F. Parlow at National Hormone and Pituitary Program) for 24 h. 24 h prior to experiments involving β-Estradiol (Sigma), media was changed to RPMI containing 5% Charcoal Stripped Serum (Thermo Scientific) and 1 mM sodium pyruvate.

### Xenotransplants

Nude mice (N = 20) were implanted with 17β-estradiol pellets (0.72 mg; Innovative Research of America) and injected subcutaneously with T47D cells (5 × 10^6^) suspended in Matrigel into two dorsolateral sites. Tumors were allowed to grow for 6 weeks and subsequently were injected subcutaneously with either saline (N = 10) or 5 μg/g body mass of human prolactin (N = 10) every 12 h for 48 h total. Tumors were harvested and processed for immunohistochemistry and qRT-PCR. All research involving mice were approved by Thomas Jefferson IACUC (protocol 789C to H.R.) in accordance with international guidelines for ethical treatment of animals.

### Microarray

RNA was pooled into 3 groups from PRL injected mice and 3 groups from saline injected mice, with each group containing RNA from 3–4 mice. Microarray analysis was performed for each group using the Affymetrix HG-U133 GeneChip Set (Expression Analysis). Two group comparison analyses were conducted on normalized expression values that were individually transformed using base 2 logarithms. On the log-transformed scale, the mean is calculated for every gene within each group and a two-sample, two-sided t-test is conducted to test the equality of those means. The P-value indicates the significance level of this test.

### Quantitative reverse transcription polymerase chain reaction

Quantitative RT-PCR assays were performed with RNA samples isolated from T47D cells using RNeasy kit (Qiagen). cDNA was generated using Iscript (Bio-Rad) according to the manufacturer’s protocol. cDNAs were subjected to qPCR using corresponding primers (Table 7).

**Table 7:** Primer sequences

**Gene**	**Forward primer**	**Reverse primer**
PTHrP	GTTCCTGGTGAGCTACGCG	CTTGGATGGACTTCCCCTTG
CCR6	TGCTACCGCTGCCTGTGAGC	AAAATAATCTTCACTGGAGTCG
IER3	CGTCCTCGAGCCCTTTAATCT	AGGTCCAGAGCGTAGTCCGA
AMIGO2	CCGGTGTCTTTTCCACCG	GAGCCCACGAGGCTCC
EREG	GCTCTGCCTGGGTTTCCATC	CCACACGTGGATTGTCTTCTGTC
ERAP1	GCCATTCTAGCTGCAGTGGG	CAACTGTGTACGGGAGCCC
DUSP4	TACAAGTGCATCCCAGTGGA	CCCGTTTCTTCATCATCAGG
CISH	CTGCTGTGCATAGCCAAGAC	GTGCCTTCTGGCATCTTCTG
TMC5	TATCCTTCAGCTCAATTGCTG	AGAGGACGCTGGTTCCAAAC
AREG	GGTGGTGCTGTCGCTCTTG	TCAGCACTGTGGTCCCCAG
FN1	TTCTACTCCTGCACACAGAAG	CCCTCAGAAGTGCAATCAGTG
NTN4	CATGGTGGGATACTGGGGC	TCAGGAACTTCATGATACCAGTC
WT1	GAGAGCCAGCCCGCTATTC	CATGGGATCCTCATGCTTG
GADD45A	TCAGCGCACGATCACTGTC	CCAGCAGGCACAACACCAC
FGFR2	CTCACTCTCACAACCAATGAGG	AGGAAGGCATGGTTCGTAAG
BCL6	CTGCAGATGGAGCATGTTGT	TCTTCACGAGGAGGCTTGAT
SOX4	GTGAGCGAGATGATCTCGGG	CAGGTTGGAGATGCTGGACTC
FLT4	CAGGATGAAGACATTTGAGG	AAGAAAATGCTGACGTAT
GAPDH	AATCCATCACCATCTTCCA	TGGACTCCACGACGTACTCA
ERAP1	GCCATTCTAGCTGCAGTGGG	CAACTGTGTACGGGAGCCC

### Immunohistochemistry

Immunohistochemistry and AQUA analyses were performed on a tissue array generated by cutting-edge matrix-assembly containing 100 deidentified primary invasive breast carcinoma specimens in a cohort described previously [33]. Immunohistochemistry was performed as described previously [33] using pY-Stat5 (Epitomics, 1:200), PTHrP (Santa Cruz, 1:200), and cytokeratin (DAKO, 1:100). AQUA analysis was performed using AQUA/PM2000 (HistoRx) as described previously [33].

### Adenoviral and lentiviral production and infection

Lentiviral particle production was performed as described previously [33]. shRNA lentiviral vectors (Open Biosystems, Lafayette, CO, USA) were obtained for scrambled control (SC002), and Jak1 (TRCN0000003102), and Jak2 (TRCN0000003180). The cells were infected with lentivirus overnight and allowed to grow for 48 h before hormone induction for an additional 24 h. LacZ, Stat5a, Stat5b, and Dominant-negative-Stat5 (DN-Stat5) adenoviruses were prepared using double cesium chloride centrifugation [53] and used for gene delivery into T47D cells (1.5×10^6^/well in 6 well dish; multiplicity of infection = 5). 24 h after infection, cells were incubated with hormones for another 24 h and subsequently harvested for immunoblot analysis.

### Cell proliferation and soft agar assays

T47D cells were treated with vehicle, PRL, or 17β-Estradiol (E2) (Sigma) for 72 h. Triplicates of each condition were plated and counted using the Countess Automated Cell Counter (Invitrogen). For the soft agar assay, T47D cells suspended in 0.3% agarose were plated on top of 0.6% agarose. T47D cells were treated with media containing either vehicle, PRL (20 nM), E2 (10 nM), or PRL + E2 for 2 weeks, with fresh media and hormones added every 3 days. Each condition was done in quadruplicate. Images were taken from each well at 2 weeks and were analyzed for colony size using ImageJ.

### Immunoblotting

T47D cells were lysed as described previously [54]. Proteins were resolved by SDS-PAGE and immunoblotted with antibodies to phospho-Stat5 (AX1, Advantex), and total Stat5 (BD #610192), followed by secondary antibodies Alexa Fluor 680-conjugated goat anti-mouse IgG (Invitrogen) or IRDye 800 CW-conjugated goat anti-rabbit IgG (Licor, Lincoln, NE, USA) depending on primary antibodies. Immunoblots were scanned using the Odyssey Infrared Imaging System (Licor).

#### Gene ontology analysis and survival analyses

The PRL-regulated genes identified using mRNA expression microarrays were analyzed for enrichment of Gene Ontology Biological Process terms using the Database for Annotation, Visualization and Integrated Discovery (DAVID ) v6.7 [55,56]. A previously described collection of mRNA microarray datasets compiled from public repositories [57] was used to evaluate the set of PRL-induced transcripts in the context of clinical outcome. Transcription levels for PRL-upregulated genes were averaged into an expression signature and used to divide samples into the lower quartile, interquartile range, and upper quartile. Relapse-free and metastasis-free survival differences in these groups were evaluated for significance using the log rank test.

## Competing interests

The authors declared that they have no competing interest.

## Authors’ contributions

TS performed all qRT-PCR experiments, knockdown experiments, proliferation assays, RNA isolation for most of the *in vitro* experiments, analysis of experimental results, creation of the figures, and drafted the manuscript. THT provided insight into study design, analysis of experimental results, and isolated RNA for the adenovirus experiments. ARP performed the adenovirus and soft agar experiments. CL performed immunohistochemistry and AQUA analysis. AE performed GO analysis and survival analyses in public mRNA data sets. JL was responsible for investigation of NTN4 and TMC5 regulation by PRL. LMN performed the T47D xenograft experiment and isolation of RNA from the xenografts. HR provided oversight, guidance and scientific input to the project and finalized the manuscript. All authors read, edited, and approved the final manuscript.
